# Analysis of Silver Nanoparticles for the Treatment and Prevention of Nucleopolyhedrovirus Affecting *Bombyx mori*

**DOI:** 10.3390/ijms23116325

**Published:** 2022-06-05

**Authors:** Boyuan Deng, Zhanqi Dong, Qin Wu, Bingyu Guo, Wenxuan Fang, Congwu Hu, Jiangqiong Long, Peng Chen, Cheng Lu, Minhui Pan

**Affiliations:** 1 State Key Laboratory of Silkworm Genome Biology, Southwest University, Chongqing 400716, China; dengboyuan99@163.com (B.D.); zqdong@swu.edu.cn (Z.D.); wuuqin@163.com (Q.W.); guo15002368720@163.com (B.G.); fang2928388155@163.com (W.F.); 18013127742@126.com (C.H.); longjqiong@163.com (J.L.); pjchen@swu.edu.cn (P.C.); 2 Key Laboratory of Sericultural Biology and Genetic Breeding, Ministry of Agricultureand Rural Affairs, Southwest University, Chongqing 400716, China

**Keywords:** AgNPs, antivirus mechanism, silkworm, effects

## Abstract

*Bombyx mori* nucleopolyhedrovirus (BmNPV) causes major economic losses in sericulture. A number of agents have been employed to treat viral diseases. Silver nanoparticles (AgNPs) have wide applications in biomedical fields due to their unique properties. The anti-BmNPV effect of AgNPs has been evaluated, however, there are insufficient studies concerning its toxicity to other organisms and the environment. We chemically synthesized biocompatible BSA-AgNPs with a diameter range of 2–4 nm and characterized their physical properties. The toxicity of AgNPs towards cells and larvae with different concentrations was examined; the results indicated a biofriendly effect on cells and larvae within specific concentration ranges. The SEM observation of the surface of BmNPV after treatment with AgNPs suggested that AgNPs could destroy the polyhedral structure, and the same result was obtained by Coomassie blue staining. Further assays confirmed the weakened virulence of AgNPs-treated BmNPV toward cells and larvae. AgNPs also could effectively inhibit the replication of BmNPV in infected cells and larvae. In summary, our research provides valuable data for the further development of AgNPs as an antiviral drug for sericulture.

## 1. Introduction

*Bombyx mori*, the silkworm, is an economically important lepidopteran insect in China that is known as a source of both edible protein and the silk used to weave its cocoons [1,2]. Many pathogens have substantial impacts on the silkworm industry. One such pathogen is *Bombyx mori* nucleopolyhedrovirus (BmNPV), a virus that causes serious annual economic losses [3]. BmNPV consists of a double-stranded circular DNA with capsid proteins that assemble a nucleocapsid structure that is wrapped in an envelope [4,5]. The interaction between BmNPV and silkworms is complex and diverse. The process of infection involves virus morphological transformation, host immune defense, viral inhibition of host immunity, host cell apoptosis, and virus integration into the host genome [6]. Different prophylactic and curative measures have been employed for controlling the disease, including the use of chemical disinfectants, antibiotics, and gene-editing techniques [7,8,9]. Antibiotics have played a critical role in treating animals infected with viruses or bacteria. However, such agents can lead to the emergence of antimicrobial resistance. Pathogenic resistance is a serious problem encountered by humans and domestic animals. With the rapid progress of molecular biology, the antiviral capacity of the silkworm has been enhanced through gene-editing technology via approaches, such as targeting viral mRNAs with RNAi [10], inhibiting BmNPV protein synthesis by hycu-ep32 overexpression [11], and suppressing BmNPV by regulating the host immune pathways [12]. Due to certain limitations of molecular technology, the silkworm industry still relies on thorough, strict breeding operation techniques and environmentally friendly disinfectants to prevent virus infections.

Silver nanoparticles (AgNPs) are nanoscale silver particles; as such, the particles possess a large surface-to-volume ratio and remarkable antimicrobial activity at low concentrations. AgNPs have become a research focus in materials science, biology, and medicine [13]. In the biomedical sciences, AgNPs are widely used for drug delivery, medical imaging, and surgical devices due to their unique properties and limited cytotoxicity against mammalian cells [14]. Currently, there is limited information concerning the mechanism of AgNPs interacting with viruses. The two aspects of the primary mechanism are as follows: (1) AgNPs bind to the outer coating of proteins, thus suppressing the attachment of the virus to cell receptors. (2) AgNPs bind to nucleic acids (DNA/RNA) and inhibit the replication or proliferation of the virus inside the host cells [15]. Biogenic AgNPs have been demonstrated to have strong antibacterial and antifungal properties, which are owing to the special mechanisms of active forms of silver’s action on pathogenic organisms [16]. In previous studies on silkworms, Li et al. confirmed that AgNPs have strong bactericidal action on Gram-positive and Gram-negative bacteria, as well as inhibition of BmNPV and BmCPV [17]. Selvaraj et al. used biosynthetic AgNPs to incubate BmNPV and showed that AgNPs attach to the surface of BmNPV, destroying the stability of the viral shell [18]. Research on the toxicity of AgNPs to the silkworm has been performed via transcriptome and proteomic analyses of the silkworm larvae after treatment with AgNPs. Results demonstrated that AgNPs can downregulate some digestive enzymes, damage the tissue of the midgut in silkworms, and in the meantime, induce the accumulation of reactive oxygen species which may cause oxidative stress [19]. The data presented herein provides valuable information on the hazards and risks of nanoparticle contamination. AgNPs have been gradually improved to become novel and efficient antimicrobial agents, although it is necessary to assess the effects of AgNPs on the host and to further explore in more detail the antimicrobial mechanism.

Based on the effect of AgNPs in the silkworm, this study further evaluated its function in resisting the infection of BmNPV and virus-host interactions. First, we synthesized and characterized AgNPs using multiple methods. Then, we selected the optimum concentration of AgNPs for silkworm treatment using several indices and further confirmed the limits of toxicity of AgNPs. An in vitro and cell culture experiment for the application of AgNPs as a potential prevention and treatment against BmNPV in silkworms was performed by using fluorescence microscopy, flow cytometry, and quantitative reverse transcription PCR.

## 2. Materials and Methods

### 2.1. Synthesis and Characterization of the Nanometer Material

The analytical pure silver nitrate was purchased from Amresco (Solon, OH, USA). Bovine serum albumin (BSA) was purchased from Beyotime Biotechnology (Shanghai, China). Unless otherwise stated, all other solutions and reagents were purchased from this company.

Silver nitrate (0.01 g/mL), 100 mL of ultrapure water, and 1 g of BSA were combined. The liquid mixture was incubated in a 100 °C water bath on a magnetic stirring plate. The pH of the solution was adjusted with sodium hydroxide to pH = 10. The silver nanoparticles were synthesized after 0.5 h of incubation when the solution changed from colorless to bronze. Then, the final solution concentration was calculated by the final volume and Ag^+^ ion content, and the solution was stored at 4 °C.

The morphology of the AgNP particles was examined by transmission electron microscopy (TEM) (JEM-1400Plus, JEOL, Tokyo, Japan). The zeta potential and size distribution of the AgNPs were analyzed by a Zetasizer Nano S90 particle analyzer (Malvern Panalytica, Malvern, UK). X-ray diffraction analysis (XRD) (D8 Advance, BrukerAXS, Karlsruhe, Germany) assessed the crystalline structure of biosynthesized AgNPs.

### 2.2. Determination of the Optimal Concentration of AgNPs

Cell states were inferred from cell proliferation and viability. The absorbance was measured using a CellTiter 96 Aqueous (Promega, Beijing, China) kit. BmE-SWU cells were stored in our laboratory. The cells (80~90% coverage) were seeded into 96-well plates (Corning, NY, USA) at an initial density of 1 × 10^5^ cells per well. AgNPs were added at varying concentrations, and the cells were cultured for 72 h. Before the absorbance was measured, the solution in 96-well plates was changed to 100 μL of TC 100-insect medium (US Biological, Swampscott, MA, USA) and 20 μL of CellTiter 96 aqueous solution to protect the culture from light damage. The absorbance associated with each well was measured by a microplate reader (Bio-Rad, Hercules, CA, USA) at a wavelength of 490 nm.

Meanwhile, we photographed treatment groups of different Ag nanoparticle densities using a fluorescence microscope (Olympus, Tokyo, Japan). The number of cells was evaluated by a cell counter (Bio-Rad).

### 2.3. Effect of AgNPs on Growth Status of the Silkworm

Silkworm larvae were obtained from our laboratory. A total of 1, 10, 25, 50 and 100 μg/mL of AgNPs were used to soak mulberry leaves once per day, and sterile water was used as a control. The 5th instar silkworm larvae were divided into 30 per group and were reared at ambient temperature (25–27 °C) on treated mulberry leaves until cocooning. We recorded the larvae weight at different time points. The above experiment was repeated at least three times.

### 2.4. Influence of Morphology and Replication of BmNPV Treated by AgNPs

BmNPV was stored in our laboratory. Different concentrations of AgNPs were incubated with BmNPV. Each treated sample was prepared by dropping the original sample onto a copper grid, followed by drying in an oven at 80 °C for 6 h.

The BmNPV was precoated with gold using an IXRF Magnetron Sputter (MSP-2S, IXRF, Austin, TX, USA) and examined using a Hitachi-SU3500 scanning electronic microscope (SEM) (SU3500, Hitachi, Tokyo, Japan). Images were viewed in the secondary electron mode (SE) at an accelerating voltage of 10 kV using a low beam current.

### 2.5. Fluorescence Observations

The NPV-EGFP constructed by our laboratory is an infection capacity baculovirus expression system that employs an enhanced green fluorescence protein (EGFP) that can be utilized for observation by a fluorescence microscope. Cells with a similar status and density were selected to be infected with NPV-EGFP, while different concentrations of AgNPs were added to the cells. The green fluorescence was observed at different time points using an Olympus FV3000 scanning electron microscope (Olympus, Tokyo, Japan) at a wavelength of 488 nm.

For the study of the antiviral activity of AgNPs, the BmE cells were infected by the same concentration of NPV-EGFP. Subsequently, 0, 1, and 2 μg/mL AgNPs were added to the culture flask. The fluorescence signal was observed using a fluorescence microscope at 0, 24, 48, and 72 h post-infection.

### 2.6. Flow Cytometry

The treated group was collected and washed twice with PBS and fixed overnight with 75% ethanol. Then, the cells were washed with PBS and incubated with RNase A and propidium iodide (PI) for 30 min at 37 °C. The cells were then analyzed using a CytoFLEX flow cytometer (Beckman Coulter, Brea, CA, USA). Flow cytometry was used to detect the percentages of FITC-positive cells in each group.

### 2.7. Real-Time Fluorescent Quantitative PCR

For further confirmation of the effect, an assessment of the BmNPV proliferation status was done by quantitative measurement of the relative *gp41* expression level (forward 5′ CCTATTCTGTGCTGGTGGTGG 3′ and reverse 5′ ATGTTGATGTGCGGAAAGC 3′). The gene *sw22934* was selected as a housekeeping gene (forward 5′ TTCGTACTGGCTCTTCTCGT 3′ and reverse 5′ CAAGTTGATAGCAATTCCCT 3′) for quantitative PCR. To identify the anti-BmNPV effect of AgNPs on the silkworm, AgNPs at 10 and 100 μg/mL were incubated with BmNPV for 30 min. The 4th instar silkworms were fed a dose of 1 × 10^5^ OBs/head, and the control group was fed untreated BmNPV at the same concentration. The status of the silkworms was observed, and the survival rate was recorded. At the same time, larvae at different processing times were chosen to measure the relative expression level of *gp41* to reflect the proliferation of BmNPV in vivo. The qRT-PCR was carried out in 10 µL reaction volumes containing 0.8 µL of template cDNA, 10 µM of F/R primer 0.3 µL, 5 μL of DEPC water, and 5 µL of 2× Hieff qPCR SYBR Green Master Mix (No Rox) in 96-well plates. The reaction program was 95 °C for 30 s, followed by 40 cycles at 95 °C for 5 s and 60 °C for 30 s. Then, a melting curve was prepared by beginning at 65 °C and increasing the temperature to 95 °C with a 0.5 °C gradient for 5 s in each step.

### 2.8. Statistical Analysis

Each experiment was repeated at least three times, and the values are presented as the mean ± standard deviation (SD). Differences between mean values were analyzed with a Student’s *t*-test. A value of *p* < 0.01 was considered statistically significant. The statistical analyses were conducted using GraphPad Prism 7 software (http://www.graphpad.com accessed on 26 November 2021).

## 3. Results

### 3.1. BSA-Synthesis of Silver Nanoparticles

**Figure 1:**
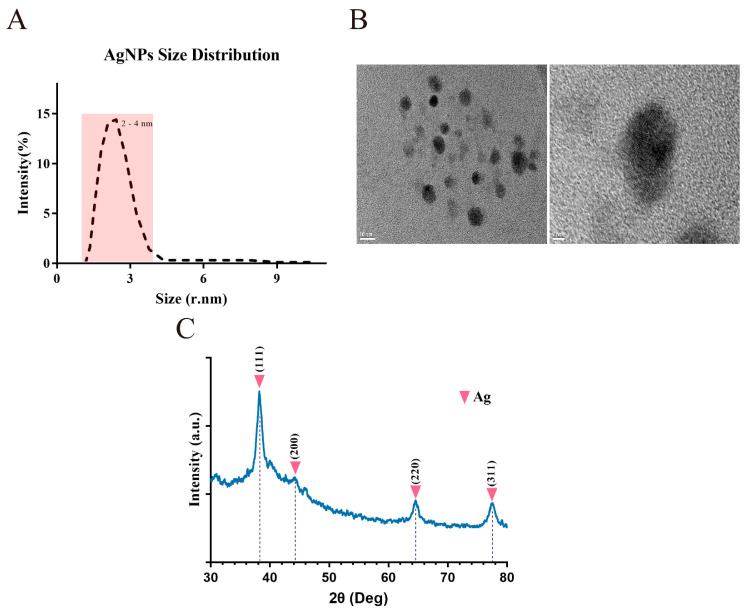
The physical properties of biosynthesized AgNPs. (**A**) Size distribution of biosynthesized AgNPs. (**B**) Transmission electron microscopy image of AgNPs. Bar scales, 5 and 10 nm. (**C**) X-ray diffraction spectrum of the biosynthesized AgNPs.

The size and structure of AgNPs determines their antiviral properties. BSA-AgNPs that were prepared in one step by the hydrothermal method present a more stable status and smaller particle size distribution regarding their physical properties. We synthesized AgNPs by using BSA as a reducing and protecting agent, and then further analyzed and tested their general properties. The TEM observations suggested that the shape of AgNPs as synthesized by BSA-AgNPs was approximately spherical, with BSA wrapped around the AgNPs core and with a relatively consistent diameter of 2–4 nm (Figure 1B). Meanwhile, dynamic light scattering (DLS) was used to calculate the size distribution of AgNPs. The analysis indicated that the size range of synthesized BSA-AgNPs was concentrated at 2–4 nm (Figure 1A). XRD patterns represent the solid phase structure and crystallinity; the four peaks of the XRD pattern were located at 2θ values of 38.1°, 46.18°, 64°, and 78.08°, corresponding to the 111, 200, 220, and 311 Bragg’s reflection, respectively, (Figure 1C), confirming the crystalline nature of AgNPs.

### 3.2. The Optimum Concentration of AgNPs In Vivo and In Vitro

**Figure 2:**
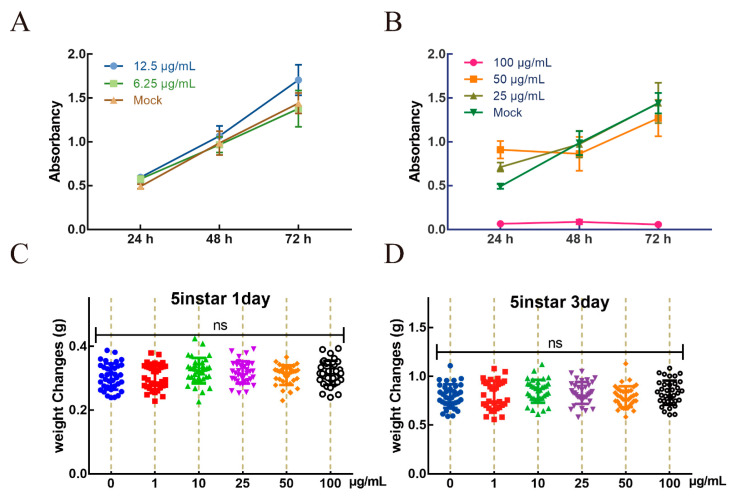
The optimum concentration of AgNPs in vivo and in vitro. Different concentrations of AgNPs fed the 5th instar larvae. Cell viability was examined using CellTiter 96 aqueous solution at low (**A**) and high (**B**) concentrations of AgNPs. Statistics of larvae weight after one (**C**) and three (**D**) days of feeding different concentrations of AgNPs-treated mulberry leaves. Different colors indicate different parallel groups. Differences in data were assessed by *t*-test, ns *p* > 0.05.

The influence of AgNPs on the silkworm is multidimensional. We first screened a concentration range of AgNPs that had little damage to silkworm BmE cells to ensure the normal state of cells in subsequent experiments. The BmE cells were incubated with five concentration ranges (1–100 μg/mL) of AgNPs, and untreated cells were set as a control. Cell viability was determined by the CellTiter 96 Aqueous at 0 h, 24 h, 48 h, and 72 h. The absorbance results showed that 12.5 μg/mL AgNPs and below had no effect on cell proliferation; after three days of treatment, the cell proliferation and viability increased significantly (Figure 2A). In contrast, cell proliferation was significantly decreased at concentrations of 25 μg/mL and above (Figure 2B). The results indicated that there was no toxic effect of 12.5 μg/mL AgNPs and below on the cells, and thus, this concentration range of AgNPs was used for further experiments.

We also confirmed the effect of AgNPs on silkworm’s growth status. The results showed that there were no significant differences between treatments and controls on silkworm weight after 1–100 μg/mL AgNPs treatment. Based on the above results, the weight of larvae after one (Figure 2C) and three (Figure 2D) days of treatment showed no significant differences between the treatment and control groups. The above results indicated that concentrations of 100 μg/mL AgNPs and below produced no side effects on the growth status of silkworms, and thus, further experiments were performed with reference to this range of AgNPs concentrations.

### 3.3. The Effect of AgNPs on Morphology of BmNPV

**Figure 3:**
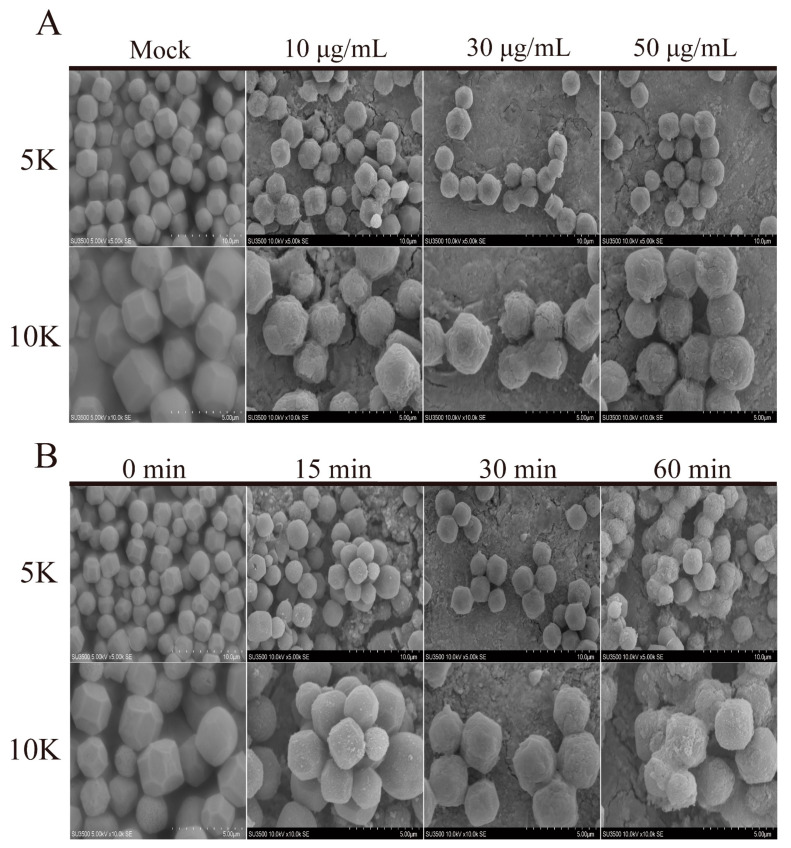
The effect of AgNPs on the morphology of BmNPV. (**A**) SEM observation of BmNPV morphology after treatment with AgNPs at different concentrations. Bar scales, 5 and 10 μm. (**B**) SEM observation of BmNPV morphology after treatment with AgNPs for different durations. Bar scales, 5 and 10 μm.

To study the effect of AgNPs on BmNPV, the morphological characteristics of BmNPV after treatment with different concentrations of AgNPs were observed by SEM. The BmNPV untreated by AgNPs were arranged regularly and exhibited a polyhedral structure of 2–4 μm in diameter (Figure 3A). The BmNPV was treated with AgNPs at concentrations of 10, 30, and 50 μg/mL for 30 min (Figure 3A). The viral envelopes began to break down, and the smooth viral surface gradually became rough; as the concentration increased, the degree of dissociation of the envelope increased significantly. We further performed a time-course study to explore the interactions between AgNPs and BmNPV. As in the initial experiment, the BmNPV showed gradual morphological changes over time at a concentration of 50 μg/mL. In the early stages, AgNPs treatment caused the polyhedrin protein to become loose; after one hour, many BmNPV had been cleaved, and the viral structure was substantially disrupted (Figure 3B).

### 3.4. AgNPs Can Inhibit BmNPV Multiplication

**Figure 4:**
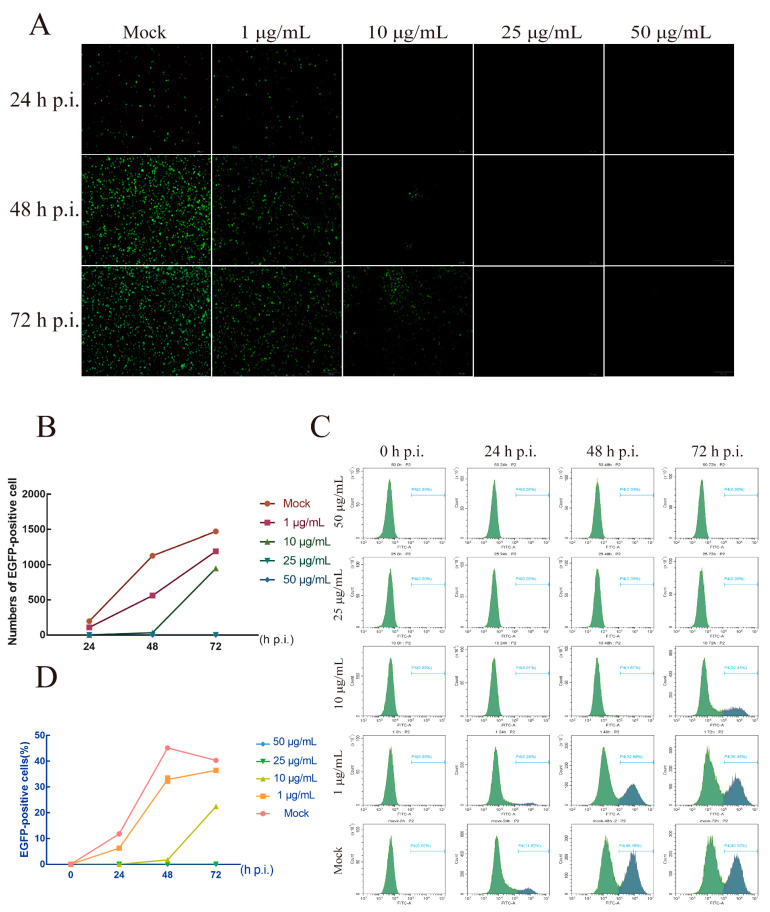
BmNPV replication could be inhibited by AgNPs. (**A**) Fluorescence microscopy image reflecting virus replication after treatment with different concentrations of AgNPs. BmNPV, green fluorescence. Bar scale, 200 μm. (**B**) Statistics for EGFP-positive cells in different treatment groups. (**C**) Flow cytometry was used to analyze BmNPV replication on BmE cells. (**D**) Statistics of the outcome of flow cytometry.

The higher concentrations of AgNPs could destabilize the envelope structure of BmNPV. Further analysis considered the effect of AgNPs on BmNPV multiplication. BmNPV was treated with AgNPs at various concentrations for various durations, and the rates of BmNPV multiplication were measured via enhanced GFP fluorescence generated by *EGFP*-expressing recombinant baculovirus (Figure 4A). After the EGFP-positive (*EGFP*+) cells were counted in the graph (Figure 4B), we quantified the rate of viral multiplication with flow cytometry (Figure 4C). In the 1 μg/mL concentration group, the number of *EGFP* signals was significantly lower than in the control at 24 h post-infection. The *EGFP*+ cell numbers also were significantly below those of the untreated group. At subsequent time points, the multiplication rate of BmNPV was slightly inhibited, while the *EGFP* signal began to emerge in the 10 μg/mL concentration group. Many *EGFP*+ cells were detected at 72 h post-infection. In addition, no fluorescence signal was observed in the higher-concentration groups, indicating that the virus could not infect cells and multiply in this higher-concentration range.

### 3.5. AgNPs as an Agent to Reduce the Infection Rate

**Figure 5:**
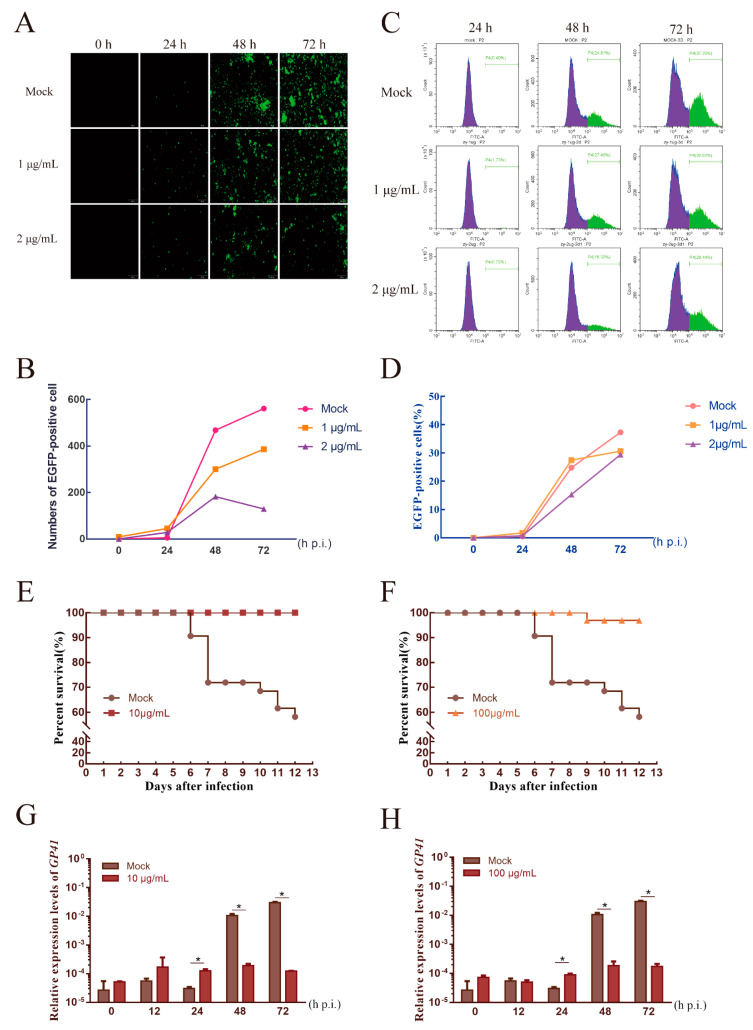
AgNPs as an agent used to reduce the infection rate. (**A**) Fluorescence microscopy images reflecting the virus replication status after different concentrations of AgNPs were used to treat the cells infected by BmNPV. BmNPV, green fluorescence. Bar scale, 200 μm. (**B**) Statistics for EGFP-positive cells in the different treatment groups. (**C**) Flow cytometry was used to analyze BmNPV replication in BmE cells. (**D**) Statistics of the outcome of flow cytometry. Survival rate analysis of silkworm larvae incubated with 10 μg/mL (**E**) and 100 μg/mL (**F**) AgNPs after infection with BmNPV. Each group replicate included 30 larvae. Each group had three replications. Viral load is reflected by the relative expression levels of GP41 after 10 μg/mL (**G**) and 100 μg/mL (**H**) AgNPs treatment in silkworm larvae. * Statistically significant difference at the level of *p* < 0.05.

The BmNPV was incubated with AgNPs and then used to infect BmE cells in order to explore the effect of AgNPs on viral multiplication. Here, we added the virus and the AgNPs at a concentration that had a minimal effect on cells to further verify whether AgNPs could reduce the viral infection rate. The fluorescence image and flow cytometry results are shown in Figure 5A,C. After the 24 h treatment, there was no significant difference between the experimental and control groups. This meant that the lower concentrations of AgNPs had little effect on the early stages of infection. However, at 48 h, the rate of multiplication was affected by the treatments; the 1 μg/mL group showed a slightly higher fluorescence signal and higher numbers of *EGFP*+ cells than the control group, whereas the 2 μg/mL group showed the opposite results. The values of the experimental groups at 72 h after treatment with each concentration were lower than those of the control group, illustrating that AgNPs could reduce the viral infection rate to some extent.

The results for the survival rate of silkworms showed the following outcomes. When the silkworms were infected by the untreated virus, there was massive silkworm mortality in the fifth and sixth days of infection. The final survival rate was 56.67%. There were no deaths in either treated group during the same time, and the survival rate of both groups was above 96% on the final day (Figure 5E,F). Based on the above treatment, we obtained silkworm samples after 0, 12, 24, 48, and 72 h post-infection, and the relative expression levels of the *GP41* gene were evaluated by RT-qPCR (Figure 5G,H). The results showed that the relative expression levels of *GP41* of both treatment groups were significantly lower than those of the untreated group at 48 h after infection. Curiously, the gene expression of the treated group was slightly higher than that of the untreated group, but the expression after that point was maintained at a low level.

## 4. Discussion

AgNP is a metallic monoplasmatic silver with a particle size in the nanometer scale, and the size is generally between 1 and 100 nm. Some studies have shown that the smaller the particle size of AgNPs, the greater the specific surface area; the contact area with bacteria will thus increase, and the antiviral effect is greatest when the particle size of AgNPs is ≤10 nm [20,21]. To ensure the antiviral effect, we adopted BSA as the packaging and protecting agent, silver nitrate as the source of Ag, and prepared BSA-AgNPs by the hydrothermal method [22]. The AgNPs materials were characterized by transmission electron microscopy, showing that the prepared AgNPs size range was concentrated around 5 nm. Some studies found that as the particle size of BSA-AgNPs decreased, the antibacterial properties were gradually enhanced, and the inhibition effect on *E. coli* was the strongest when the particle size was around 5 nm [23,24]. The combination of the XRD pattern and absorption peak results indicated that the AgNPs had desirable properties and served as a stable material in antiviral experiments.

The progress of viral infection is regulated by complex interactions between the virus and the host cell. All viruses rely on host cells to synthesize their necessary proteins. The virus must bind to the cells and inject its genome into the host cytoplasm [6]. Therefore, eliminating the virus in vitro or preventing it in the early stages of infection are key strategies employed to alleviate viral diseases. Many studies have shown that the antiviral effect of AgNPs is produced through these strategies. Hsueh et al. [25] demonstrated that AgNPs could efficiently inhibit the growth of Gram-positive *B. subtilis* bacteria but were toxic to cells by damaging cellular membranes, degrading chromosomal DNA, and reducing a series of protein activities. In another study, the continuous action of AgNPs significantly inhibited and alleviated H1N1 influenza infection in a mouse model. The antiviral mechanism relies on direct-acting antivirals for the virus and indirectly acting antivirals for the host [26]. The *Bombyx mori* nuclear polyhedrosis virus replication cycle includes two virion phenotypes during replication: a budded virus (BV) and an occlusion-derived virus (ODV). The ODV is embedded in the polyhedron and is released after cell disintegration, causing primary infection in host individuals [2,27]. Polyhedrons form solid crystal particles to wrap the virus particles. The coated polyhedron can protect virus particles from a variety of external factors so that the virus can maintain stability and infectivity in a harsh environment. A series of studies demonstrated that treatment of BmNPV with AgNPs could significantly reduce the incidence rate of viral disease [28]. Selvaraj et al. used microscopic observations to show that AgNPs accumulated on the surface of BmNPV, and some AgNPs penetrated the nucleocapsid. The resulting destruction would make the virus lose stability [18]. It was observed that with increases in concentration and time; the BmNPV was gradually destroyed, and the normal virus structure was disrupted. At the same time, the mortality from BmNPV incubated with AgNPs decreased from 100% to 60%, and the virus load also decreased significantly, indicating that AgNPs can cause certain structural damage to the polyhedron and the ODV can be released in advance and damaged, reducing the ability to infect the silkworm. The effect of AgNPs on viral polyhedrons was further examined, and significant degradation was observed (Figure S1). AgNPs destroyed the viral structure, demonstrating that AgNPs treatment could damage the viral protein shell to decrease infectivity in the early stages.

Some specific active drugs have been evaluated for their antiviral activity. The bacterial secondary metabolite prodigiosin can inhibit BmNPV in BmN cells by selectively killing virus-infected cells, thereby inhibiting viral replication at very early stages and preventing virus-mediated membrane fusion [29]. PI3K/Akt pathway inhibitors can inhibit induced p-Akt to prevent the proliferation of BmNPV in BmE cells [30]. BV, another virus particle form produced in the replication of BmNPV, is released by budding from the plasma membrane to neighboring cells, resulting in systemic infection [31]. We used particles of homologous recombinant EGFP protein to explore the interaction between BmNPV and AgNPs and its effect on virus proliferation. After BV particles were incubated with a certain concentration of AgNPs, the infection rate and replication ability of BmNPV were significantly inhibited. In contrast, AgNPs with no effect on the concentration of cells were added to the cells infected with BV particles. With the extension of incubation time, the BV particles with green fluorescence gradually decreased, indicating that AgNPs can play a role in the treatment of BmNPV. While paying attention to the significant effects of these agents, we should also consider the risks to the host organism and the environment. Prodigiosin has not been validated concerning its effect in vitro, and the toxic effect on individual silkworms is unknown. The absorption and utilization efficiency, inhibition effect, and cost of PI3K/Akt pathway inhibitors need to be further optimized. More research into the effects of AgNPs is needed as well as in vitro and in vivo studies to better understand the severity of negative effects of AgNPs and optimize the application method. The proliferation and viability of BmE cells treated with different concentrations of AgNPs were measured, and the morphological structure of the cells was concurrently observed by microscopy. We find that at concentrations above 12.5 μg/mL, the AgNPs affected the normal growth of BmE cells, while concentrations of less than 5 μg/mL promoted cell proliferation and viability, with little effect on cell growth [32]. In this study, different concentrations of AgNPs were used to treat mulberry leaves with the soaking method. When the silkworms were fed the treated leaves at the 5th instar, 100 μg/mL or less did not significantly affect silkworm larval weight, which is consistent with our previous results that the cocoon weight, cocoon shell weight, and cocoon shell rate of each group of AgNPs-treated silkworms were not significantly different from those of untreated silkworms, and the egg-laying ability of the moths was approximately the same as that of the normal group [33].

At present, the use of disinfectants is standard practice in the application of antiviral drugs for silkworms, however, there is still widespread use of oral drugs [34]. AgNPs have efficient and comprehensive anti-BmNPV performance. They can be used, however, not only as disinfectants to inhibit a variety of pathogens of silkworms but also as oral drugs to treat silkworm viral diseases. Additional research is needed to systematically explore the effect of AgNPs on other silkworm pathogens, however, such research also needs to consider the potential risks to the environment and the host organisms. Our results show that AgNPs can effectively inhibit the proliferation of BmNPV and treat Bombyx mori infected with BmNPV.

## Data Availability

The data can be accessed from the corresponding author.

## Supplementary Materials

The following supporting information can be downloaded at: https://www.mdpi.com/article/10.3390/ijms23116325/s1.
