# Supplementary material for: Analysis of Silver Nanoparticles for the Treatment and Prevention of Nucleopolyhedrovirus Affecting Bombyx mori
Source: Int J Mol Sci. 2022 Jun 5;23(11):6325. doi: 10.3390/ijms23116325 (PMC9181153; doi:10.3390/ijms23116325)
Supplement: Supplementary file 1 [file ijms-23-06325-s001.zip › ijms-1718445-supplementary.pdf]

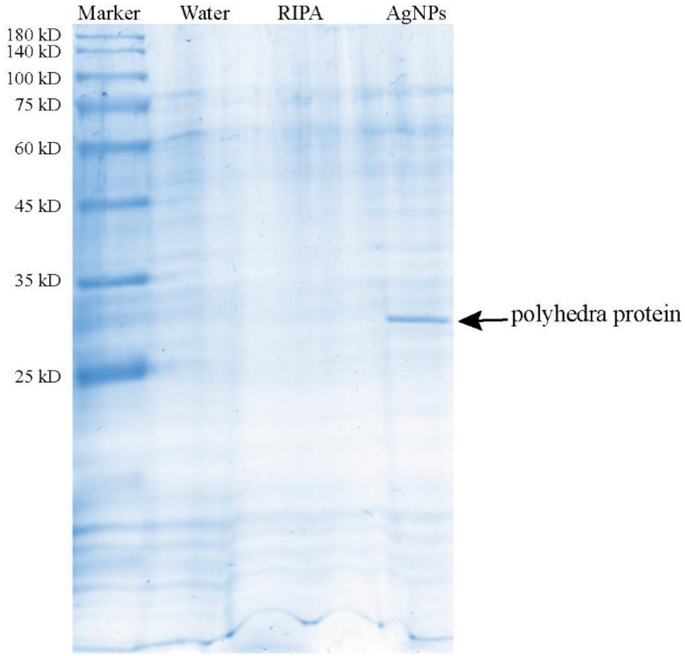

Figure S1. AgNPs affected the protein structure of BmNPV. Coomassie blue staining show BmNPV treated with a different lysis buffer.
